# Pyrethroid neurotoxicity studies with bifenthrin indicate a mixed Type I/II mode of action†

**DOI:** 10.1002/ps.5300

**Published:** 2019-01-30

**Authors:** Derek W Gammon, Zhiwei Liu, Appavu Chandrasekaran, Shaaban F El‐Naggar, Yuri A Kuryshev, Sharon Jackson

**Affiliations:** ^1^ FMC Agricultural Solutions Global Regulatory Sciences Ewing NJ USA

**Keywords:** bifenthrin, pyrethroids, Type I and II, voltage‐gated Sodium Channel, mode‐of‐action, ion channels

## Abstract

**BACKGROUND:**

Bifenthrin is usually considered a Type I pyrethroid, because it lacks an α‐CN group present in Type II pyrethroids, but some previous studies suggest a mixed Type I/II mode‐of‐action. Results are presented for bifenthrin in a rat developmental neurotoxicity (DNT) study along with effects on Na currents in human VGSC subtypes. Molecular modeling comparisons were also made for bifenthrin and other pyrethroids.

**RESULTS:**

In a rat DNT study, bifenthrin produced tremors and clonic convulsions in dams and pups and slightly reduced acoustic startle response amplitude, and increased Tmax, at PND20 in females. Similar blood levels of bifenthrin were measured in dams and pups at each dose level i.e. no concentration in pups. In human VGSC experiments, using the Nav1.8 subtype, bifenthrin's effects on inactivation were slight, as for Type II pyrethroids, but without large prolongation of the tail current (deactivation) seen with Type II. Molecular modeling of bifenthrin indicates that the *o*‐Me group may occupy a similar space to the α‐CN group of cypermethrin and fenpropathrin.

**CONCLUSION:**

In a DNT study and on human Nav1.8 tail currents bifenthrin showed Type I and II effects, similar to some published studies. Overall, bifenthrin acts as a mixed Type I/II pyrethroid. © 2018 The Authors. *Pest Management Science* published by John Wiley & Sons Ltd on behalf of Society of Chemical Industry.

## 1 INTRODUCTION

The synthesis of the first synthetic pyrethroid insecticide, allethrin, was based on the structure of pyrethrin I, a component of pyrethrum extract isolated from Chrysanthemum plants. The synthesis of modern pyrethroid insecticides, in which many of the sites of photochemical and metabolic attack had been stabilized, was pioneered by Michael Elliott and colleagues in the 1960–1980 period.^1^ Starting in the early 1960s, Toshio Narahashi studied the mechanism of action of pyrethroids (allethrin), using giant axons from the cockroach.^2^, ^3^ Giant axons from the squid and crayfish were subsequently studied in efforts to understand the interaction of allethrin with the voltage‐gated sodium channel (VGSC).^4^, ^5^, ^6^ At low temperatures, allethrin reduced sodium ion conductance leading to nerve blockage, whereas at high temperatures, delayed sodium channel inactivation and increased negative after‐potential resulted in repetitive firing following electrical stimulation. Pyrethroids are generally more toxic to insects as temperature is reduced^7^, ^8^, ^9^ and so it was hypothesized that nerve blockage was more critical than nerve hyper‐excitation.^5^

**Figure 1:**
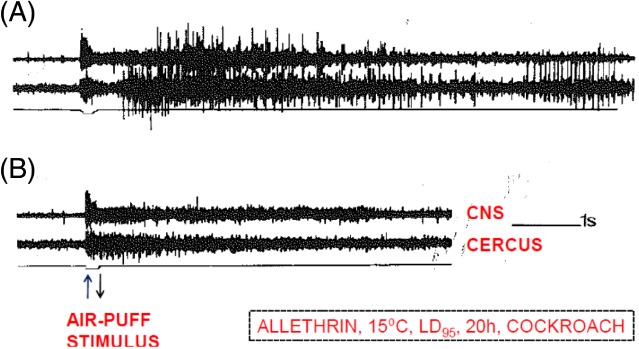
Recordings taken from the abdominal nerve cord (CNS) and a cercal nerve in an intact electrode‐implanted cockroach, 20 h after dosing with a LD_95_ dose of allethrin at 15 °C. A weak twitch of a leg was observed following a brief mechanical stimulus (air‐puff) of the cerci in A and no movements were observed after a second air‐puff 30 s later in B. Adapted from Reference 8.

In the 1970s, efforts were made to establish whether other nerves behaved similarly to giant axons with respect to allethrin action.^7^, ^8^, ^9^ In the free‐walking, electrode‐implanted cockroach it was found that this was true, but only at a relatively high temperature (32 °C). Here, the CNS and cercal sensory nerves both fired repetitively following stimulation after dosing at the LD_95_ level. However, at 15 °C, a LD_95_ dose of allethrin (approximately 10% of that at 32 °C) gave rise to repetitive firing only in cercal sensory nerve fibers; repetitive discharges and nerve blockage in the CNS at 15 °C were shown to be secondary effects (Fig. 1). The VGSCs in the cockroach cercal sensory nerves are very sensitive to blockage by tetrodotoxin or TTX.^10^ Reversible blockage of sensory axons by TTX in the cockroach blocked the nerve excitatory and lethal effects of allethrin at 15 °C.^9^

Subsequently, a study was conducted on the effects of two newer pyrethroids, permethrin and cypermethrin, on the Egyptian cotton leafworm, *Spodoptera littoralis*.^11^, ^12^ The *in vitro* responses of the abdominal nerve cord to permethrin were similar to those of allethrin on giant axons in the cockroach nerve cord i.e. repetitive firing following stimulation, but only at high temperatures. Cypermethrin had qualitatively different effects, with no repetitive firing over a range of temperatures, and a range of higher concentrations. A strain of *S. littoralis* showed resistance to permethrin, due to reduced nerve sensitivity, but not cypermethrin and it was concluded that a qualitative difference was responsible for this resistance profile. Studies using a variety of pyrethroids, without and with an *alpha* CN group, established that the effects, in rodents and insects, of allethrin, permethrin and a number of other unsubstituted pyrethroids were similar and were considered Type I effects. In contrast, effects of cypermethrin and a number of other *alpha* CN substituted pyrethroids were also similar and termed Type II effects.^13^, ^14^, ^15^ Thus there was consistency between studies on clinical signs in the rat and mouse and on clinical signs and neurophysiological effects in insects.

Bifenthrin (FMC 54800, CAS# 82657‐04‐3) is a pyrethroid that was developed in the early/mid‐1980s, and was not included in the early studies defining Type I and II pyrethroids. Because it lacks an α‐CN group, a critical feature of Type II pyrethroids, bifenthrin is often considered to be a Type I pyrethroid. Studies described here include a DNT study designed to establish whether or not young rats were more sensitive than adults in a dietary study where pups were exposed *via* lactation and one using human VGSCs *in vitro*, to assess bifenthrin effects on currents compared with effects of Type I and II pyrethroids. Molecular modeling was used to compare the structure of bifenthrin with Type I and II pyrethroids. Literature reports considered in the assessment of bifenthrin mode‐of‐action include assessments of bifenthrin effects on the cockroach VGSC, clinical signs in mammals, *in vitro* effects on the rat and human Nav1.8 subtype and bifenthrin's effects described in USEPA studies on rodent VGSC assays, *in vitro*.

## 2 MATERIALS AND METHODS

### 2.1 Rat developmental neurotoxicity study (USEPA 870.6300 and OECD 426)

Four groups of mated female SD rats (n = 25/group) were dosed by diet with 0, 50, 100 or 125 ppm (mg kg^−1^ diet) bifenthrin continuously from gestation day 6 (GD6) to lactation day 21 (LD21).^16^ The study satisfied good laboratory practices according to guidelines. All rats were examined twice daily for appearance and behavior. Body weights, food consumption and clinical signs were recorded at suitable intervals. Also, functional observational battery (FOB) assessments were made on dams at GD10 and GD15, as well as LD10 and LD21. Dams were allowed to deliver and rear offspring until LD21. At postnatal day 4 (PND4), litters were standardized to eight pups/litter by culling excess pups. At least 3/sex/litter were obtained wherever possible. After culling, subset A consisted of 20 pups/sex/dose group assigned at random to FOB (PND4, 11, 21, 35, 45 and 60), acoustic startle response or ASR (PND20 and 60), motor activity (PND13, 17, 21 and 61) and learning/memory (from PND62) assessment. ASR responses (Vmax) were measured during the first 100 ms after the start of a 20 ms ASR stimulus (115 ± 5 dB mixed frequency noise burst). Each test consisted of 50 trials (five blocks of 10) with an 8 s inter‐trial interval. Vave is the average of five blocks of 10 trials. Also, Tmax was measured as the mean time from the onset of ASR stimulus to the peak of ASR response. Subset B of 20 pups/sex/dose group was assigned at random to learning/memory (initiated on PND25) assessment. Subset C consisted of 15 pups/sex/dose group assigned at random to brain weight measurements on PND21. Of these, 10/sex were selected, at 0 and 125 ppm, for neuropathological and morphometric evaluations. Individual body weights were recorded on PND1, 4, 7, 13, 17 and 21 and weekly thereon. All pups not evaluated in these assays were subjected to full necropsy on PND21, as were Subset B animals at PND25. In a pilot study^17^ at the same doses, bifenthrin concentrations were measured in dams' milk and plasma as well as in pups' plasma, at several time points i.e. LD4 and 22 (dams) and PND 4 and 22 (pups).

### 2.2 Bifenthrin effects on the human VGSC Nav1.8/β3 in CHO cells

**Figure 2:**
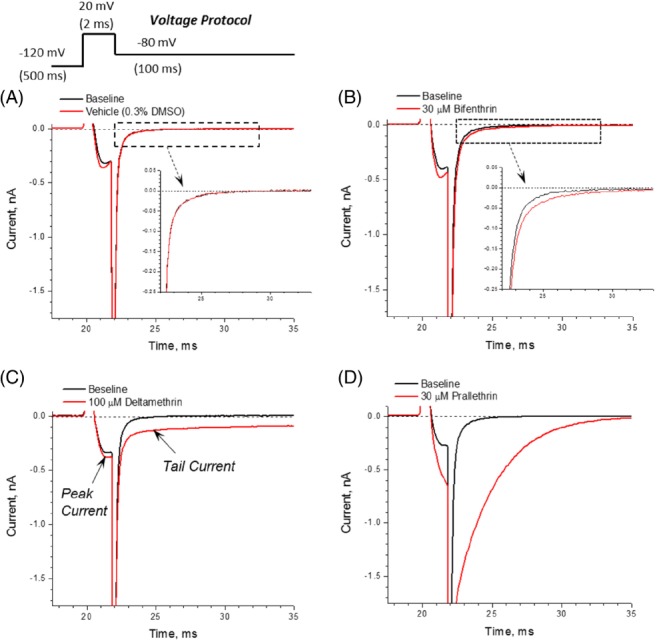
Pyrethroids modify deactivation of hNav1.8/β3 VGSC. Representative current traces recorded in CHO cells expressing hNav1.8/β3 VGSC. Control currents (a), effect of bifenthrin (b), deltamethrin, Type II (c) and prallethrin, Type I (d). Mean tail current amplitudes were measured at 23–33 ms interval. Detailed methods are provided in the Appendix S1 file.

Pyrethroid effects on human VGSC Nav1.8/β3 expressed in Chinese hamster ovary (CHO) cells, were measured using an automated patch clamp system. CHO cells (ATCC, Manassas, VA) were transfected with cDNA for specific VGSC α‐isoforms (hNav1.1, 1.2, 1.3, 1.4, 1.5, 1.6, 1.7 and 1.8/β3). Cells were cultured in Ham's F‐12 supplemented with 10% fetal bovine serum, 100 U/mL penicillin G sodium and 100 mg mL^−1^ streptomycin sulfate. Before testing, cells in culture dishes were washed twice with Hank's balanced salt solution treated with accutase and re‐suspended in HB‐PS. Immediately before use in the IonWorks Barracuda™ system (Molecular Devices Corp., Union City, CA), cells were washed twice in HB‐PS to remove accutase and re‐suspended in HB‐PS. A 384‐well plate was used to receive cells with a total volume of 40 µL. The well wall served as the extracellular Population Patch Clamp™ (PPC) planar electrode. After the establishment of a whole‐cell configuration (the perforated patch), membrane currents were recorded using the patch‐clamp amplifier in the IonWorks Barracuda™ system. Two sets of current recording were made, before and 5 min after test chemical application, the optimal time to evaluate pyrethroid effects in pilot studies using allethrin and other VGSC activators/blockers. Eight concentrations of each chemical were assessed (0.03 to 100 µm). Seven pyrethroids considered were cypermethrin, bifenthrin, pyrethrum, prallethrin, permethrin, esfenvalerate and deltamethrin. In addition, control chemicals used for validation purposes were the Type I pyrethroid allethrin at 100 µm (agonist), veratridine at 300 µm (agonist) and TTX at 0.2 µm (inhibitor). The test article vehicle was DMSO (0.3%). Extracellular buffer (HB‐PS) contained HEPES (10 mm), NaCl (137 mm), KCl (4 mm), CaCl_2_ (1.8 mm), MgCl_2_ (1 mm), glucose (10 mm), adjusted to pH 7.4 with NaOH. Intracellular solutions for whole cell recordings contained CsF (90 mm), CsCl (50 mm), MgCl_2_ (5 mm), EGTA (1 mm), HEPES (10 mm), adjusted to pH 7.2 with KOH. The voltage protocol used for assessing pyrethroid effects on activation, inactivation and deactivation (tail current decay) is shown in Figure 2: from a holding potential of −120 mV, a 2 ms depolarization to +20 mV was applied, followed by a return to −80 mV. For assessing use‐dependent modification of channel dynamics, trains of 20 depolarizations at 10 Hz. (−80 to 0 mV) were applied and currents measured.^18^ This 20‐pulse protocol was optimal for evaluating pyrethroid effects in pilot studies. Experiments were conducted at room temperature (21–22 °C). More details are provided in Appendix S1.

Three‐dimensional renderings of the pyrethroids were generated using MarvinSpace version 15.1.19.0 within ChemAxon MarvinSketch software

## 3 RESULTS

### 3.1 Bifenthrin effects in the rat developmental neurotoxicity study

This study was designed to determine whether or not pups were more sensitive to bifenthrin than adults following dietary exposure of dams. Effects of bifenthrin on the Acoustic Startle Response (ASR) were assessed on pups at PND20 and also at PND60, at which point the pups had been consuming undosed diet (diet without bifenthrin) for approximately 40 days following their lactational exposure from PND1 to 20 (Table 1). PND20 female pups exhibited a numerically decreased ASR response (V_max_) in terms of magnitude at 100 and 125 ppm (n.s.), but not at 50 ppm, compared with control. Female pups also showed an increase in T_max_ (the time in ms from the onset of the eliciting stimulus to the peak of the ASR response) at 100 and 125 ppm (both *P* < 0.05) but not at 50 ppm. These effects had resolved by PND60, showing reversibility of these neurotoxic effects on ASR. Bifenthrin did not cause any effects on ASR amplitude or latency in male rats suggesting that the ASR effects were weak at 100 and 125 ppm.

**Table 1:** Bifenthrin effects on the ASR in rat pups at PND20 and PND60 in a rat developmental neurotoxicity study.^16^

	Females†				Males†			
Parameter	0 ppm	50 ppm	100 ppm	125 ppm	0 ppm	50 ppm	100 ppm	125 ppm
Vmax								
PND20‡	129	148	106 (18%↓)	117(9.3%↓)	107	113	91	114
PND60	69.2	82.7	63.7	78.6	102	124	125	140
Vave								
PND20	23.6	28.8	20.5 (13%↓)	21.9(7.2%↓)	19.8	22.3	17.1	21.5
PND60	10.8	14.5	9.8	13.2	19.0	23.8	22.6	26.6
Tmax, msec								
PND20	23.6	23.6	26* (13%↑)	25* (5.9%↑)	26.9	25.3	28	26.6
PND60	31.5	32.0	33.0	33.3	31.5	31.1	29.9	32.5

* *P* < 0.05.

† N = 20 rats/dose.

‡ Dams were dosed with bifenthrin until PND21.

Groups of dams were given diets containing 50, 100 or 125 ppm bifenthrin until PND 20. Effects given are on maximum magnitude of effect (V_max_), average magnitude of effect (V_ave_) and time to peak effect after stimulation (T_max_). Lower sensitivity settings were used to measure ASR in adults than pups.

Mean plasma concentrations of bifenthrin were 0.13, 0.24 and 0.33 mg L^−1^ in dams at LD4, 0.13, 0.23 and 0.30 mg L^−1^ in dams at LD22 and 0.11, 0.19 and 0.24 mg L^−1^ in pups at PND4 and 0.11, 0.18 and 0.13 mg L^−1^ in pups at PND22 i.e. no signs of concentrating in pups *vs*. dams.^17^ Clinical signs (tremors) were not recorded in dams on GD10 or GD15 but they were recorded at 125 ppm during lactation. At 100 ppm, at LD21 but not at LD10, 3 of 23 dams showed slight body tremors and 2 of 23 had clonic convulsions. At 125 ppm, at LD10, 7 of 25 dams showed tremors (*P* < 0.01) and at LD 21, 13 of 25 (*P* < 0.01) displayed them and 10 of 25 showed clonic convulsions (*P* < 0.01). For male pups, tremors/convulsions were absent at PND4, 11, 35, 45 and 60, but on PND21, at 125 ppm, 4 of 20 males showed slight tremors and clonic convulsions (limbs). At PND21 rats would have been exposed to bifenthrin *via* both milk and diet. These signs were not observed in female pups. Furthermore, no bifenthrin‐related effects were observed on learning, memory or swimming ability in the Biel Maze Test.^16^ Similarly, bifenthrin did not cause developmental toxicity or nerve histopathology in the DNT study.

Thus, the dose of 50 ppm of bifenthrin was a NOEL for both dams and pups for neurotoxicity. At 125 ppm, dams had a higher incidence than pups of tremors/ convulsions; this correlates with higher blood levels in dams than pups (0.30 *vs*. 0.13 mg L^−1^ at LD/PND 22) so it may not reflect greater PD sensitivity of dams than pups. A clear maximum tolerated dose of 125 ppm was attained in this study.

### 3.2 Bifenthrin effects on the human VGSC Nav1.8/β3 in CHO cells

Figure 2(b) (arrow) shows the effects of bifenthrin, compared with solvent control (Fig. 2(a)), on human Nav1.8/β3 channels expressed in CHO cells under patch‐clamp conditions. At 30 µm, bifenthrin caused slight effects on inactivation and deactivation, upon repolarization of the membrane. Deltamethrin (Type II) at 0.1 mm, Fig. 2(c), also caused a slight effect on inactivation, similar to bifenthrin, but a large prolongation of the tail current (deactivation). Prallethrin (presumed Type I) at 30 µm caused large increases in inactivation and tail current magnitude (Fig. 2(d)).

**Figure 3:**
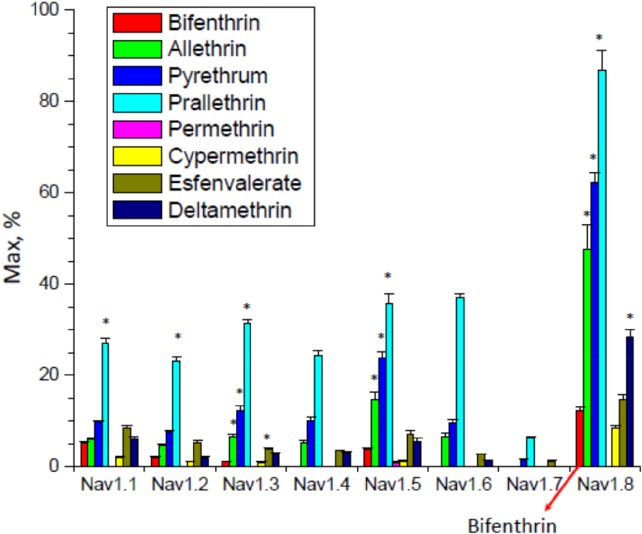
The efficacy of eight pyrethroids, including bifenthrin (marked for Nav1.8), on tail current amplitude of eight human VGSC isoforms shown as a percentage of maximum amplitude, using a patch clamp technique. Data are plotted as Max fitting parameter value and Standard Error (fitting error). * indicates 3× difference from bifenthrin Max value.^18^

The efficacies of (8) pyrethroids on tail current amplitude are given in Figure 3, in eight different human VGSC isoforms, with Nav1.8 on the right. Efficacy refers to the maximum percentage increase in tail current amplitude obtained with each pyrethroid. The largest tail currents were obtained with Type I pyrethroids, such as prallethrin, pyrethrum and allethrin, thus showing significantly higher efficacy than bifenthrin. Although showing prolonged tail currents, Type II pyrethroids, such as cypermethrin and esfenvalerate, resulted in more modest increases in tail current amplitude over the control, similar to bifenthrin (arrow in Fig. 3).

**Figure 4:**
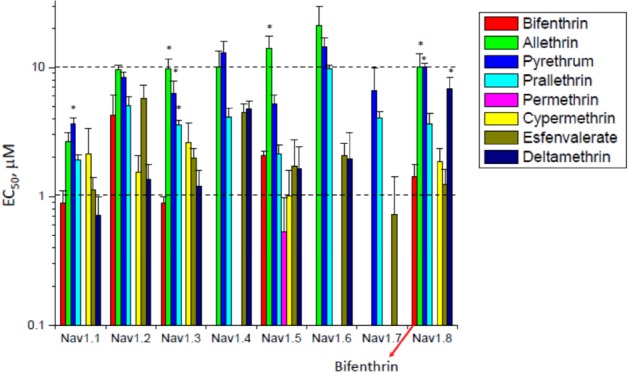
The potency (EC_50_) of eight pyrethroids, including bifenthrin (marked for Nav1.8), for effects on tail current amplitude of eight human VGSC isoforms, using a patch clamp technique. Data are plotted as EC_50_ fitting parameter value and Standard Error (fitting error). For IWB/Nav1.x assays the minimal significant ratio (MSR) is equal 4.0 (Charles River Laboratories validation data) and EC_50_ values could be considered as significantly different when their ratio is ≥4.0. *indicates significant difference from bifenthrin.^18^

The relative potency (EC_50_) of these pyrethroids, or sensitivity of the channel tail currents, is given in Fig. 4. With one exception (permethrin, which had little/no effect), the pyrethroids had EC_50_ values between 1 and 10 µm against Nav1.8. Type I pyrethroids (allethrin, pyrethrum, prallethrin) had relatively low potencies, with EC_50_ values close to 10 µm, whereas Type II pyrethroids (cypermethrin and esfenvalerate) had higher potencies, with EC_50_ values close to 1 µm. Bifenthrin (arrow) clustered with the Type II pyrethroids, with an EC_50_ between 1 and 2 µm. However, deltamethrin (Type II) had an EC_50_ closer to 10 µm. These data show that bifenthrin may be closer to Type II pyrethroids than Type I pyrethroids in terms of efficacy and potency of pyrethroids on tail currents in the human Nav1.8 channel.

### 3.3 Chemical structures and molecular modeling of bifenthrin, cypermethrin, fenpropathrin and permethrin

**Figure 5:**
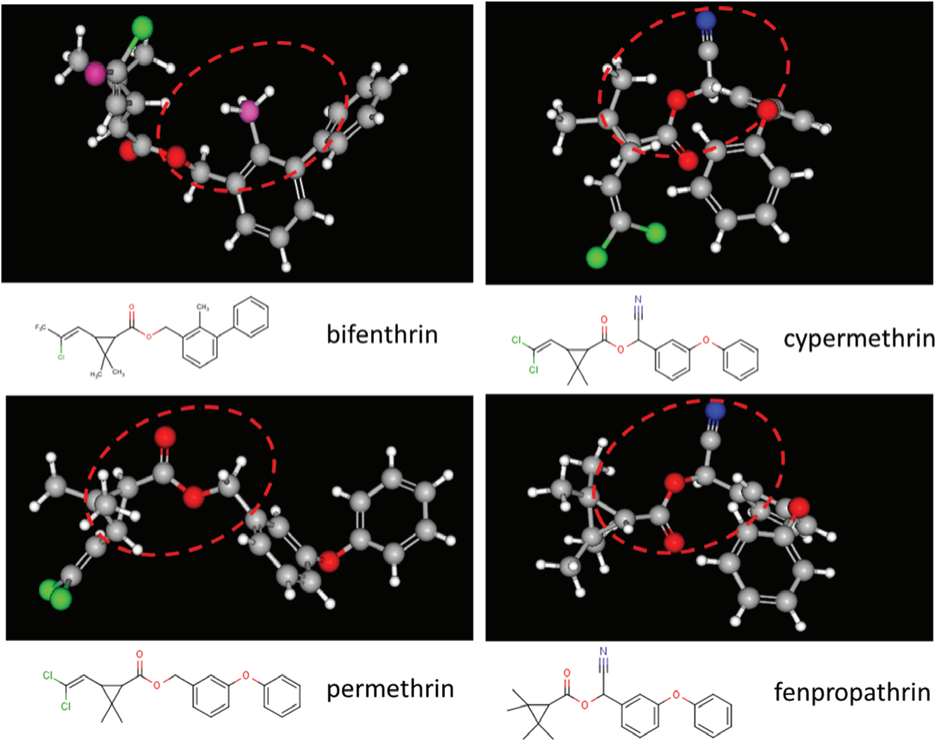
3D representation of Bifenthrin, Cypermethrin, Fenpropathrin and Permethrin. Dashed lines mark the *o*‐Me group of bifenthrin, the *α‐*CN group of cypermethrin and fenpropathrin, and the methylene group of permethrin.

A comparison of the 3D structures of these pyrethroids, in their lowest energy conformations, shows that the *o*‐Me of bifenthrin can occupy a similar space to the α‐CN of cypermethrin and fenpropathrin (Fig. 5). This structural similarity may present a plausible explanation for the mixed pyrethroid effects shown by bifenthrin.

Pyrethroids QSAR studies have shown that minimum required chemical structure domain for pyrethroid bioactivity, is encompassed within the [dimethyl‐cyclopropyl (or isopropyl‐C)—(C=O)—O] moiety of chrysanthemic acid *esterified* to a benzylic C‐connected to alcohol moiety system possessing a contiguous series 6–7 unsaturated carbons system.^19^

Folding the molecular structure of cypermethrin, bifenthrin and fenpropathrin, to its most thermodynamically stable form, would result in a similar Spatial Steric Puckering of either the α‐cyano‐function (blue) group or the 2‐methy‐phenyl group atop the ‘planar’‐ester function domain (—(C=O)—O) between the chrysanthemic acid and the alcohol system of Ring‐A.

It is hypothesized that as bifenthrin Ring‐A spins around the benzylic carbon axis, the 2‐methyl‐phenyl group of Ring‐A would share the same steric space domain as that of the α‐cyano‐function of cypermethrin. This would provide similar steric folding around the active site domain and hence similar interactions at the biological receptor binding site exerting pyrethroid Type II effects.

In the case of permethrin, the structure folds into its most thermodynamically stable form, an unoccupied spatial steric space in the domain above the ‘planar’ ester function (—(C=O)—O) region between the chrysanthemic acid and the benzyl alcohol system of Ring‐A. Hence, the resulting conformation would exhibit a slightly different interaction at the target biological receptor binding site domain, resulting in a slightly different pharmacodynamics conventionally known as the Pyrethroid Type I effect.^13^, ^14^, ^15^

Fenpropathrin, which is considered as a Type II pyrethroid, because it contains the α‐CN group, clusters with bifenthrin in some of the pyrethroid mode of action assays. Therefore, fenpropathrin has been considered as a mixed Type I/II pyrethroid.^13^, ^14^, ^15^

## 4 DISCUSSION

Since the seminal work on allethrin mode of action on nerve conduction by Toshio Narahashi was published, in 1962, a great deal has been written about pyrethroid insecticide mode of action. Many of these studies were conducted *in vitro* on nerve or channel preparations. Other studies have used *in vivo* systems in an effort to correlate *in vitro* effects with *in vivo* outcomes.

Multiple studies have been conducted on pyrethroid mode of action implicating the VGSC as the principal target site.^2^, ^3^, ^4^, ^5^, ^6^ Multiple assay systems, including *in vitro* and *in vivo* studies in both insects and mammals, have shown in most animal models two main modes of action with only a small number of exceptions that may show both sets of syndromes.^13^, ^14^, ^15^, ^20^ In the rat, choreoathetosis (sinuous writhing) with salivation (CS syndrome) was reported after intraperitoneal injection of α‐CN containing pyrethroids and tremors for non α‐CN containing pyrethroids (T syndrome).^13^ In the cockroach, prolonged cercal motor discharges unrelated to stimulation, with a lack of repetitive firing in cercal sensory nerve axons, were associated with convulsions for CS pyrethroids (Type II) whereas prolonged discharges in cercal sensory nerve axons were associated with hyperactivity for T pyrethroids (Type I).^14^, ^21^ Two types of clinical signs in intracerebrally‐injected mice correspond to Type I and II pyrethroids in the cockroach.^15^ Although pyrethroids are very toxic by intracerebral injection, this does not necessarily mean that the brain is the only or the principal pyrethroid target site. Several pyrethroids developed since the early 1980s, including bifenthrin, were not included in these original studies. However, bifenthrin was found to lack the ability to cause Type I discharges in cockroach sensory axons^22^ and it was also found to cause effects that were neither clearly Type I or Type II in multiple systems.^23^, ^24^, ^25^ Table 2 shows pyrethroid effects on rat Nav1.8 (adapted from Choi‐JS and Soderland).^24^ Bifenthrin effects on time constants for activation and deactivation are similar to Type I pyrethroids, whereas effects on inactivation are similar to Type II pyrethroids. In the current report, the effects of bifenthrin on other systems are reported for the first time and the results are compared with those reported in earlier studies.

**Table 2:** Kinetics of activation, inactivation and tail current decay (deactivation) for pyrethroid‐modified rat Nav1.8 VGSCs expressed in *Xenopus* oocytes (redrawn from Ref.25)

	Activation†		Inactivation‡		Tail current decay§		
Compound*	N	t_act_	N	t_inact_	N	τ_1_	τ_2_
Allethrin (I)	2	3.9 ± 0.8	2	21.8 ± 1.1	2	1.9 ± 0.4	–
Cismethrin (I)	9	3.3 ± 0.1	9	21.4 ± 2.0	5	2.6 ± 0.2	–
Permethrin (I)	3	2.5 ± 0.5	3	39.8 ± 19.4	3	2.3 ± 0.3	–
Deltamethrin (II)	5	15.7 ± 2.1	–	nd**	3	5.3 ± 1.7	52.1 ± 11.0
Cypermethrin (II)	4	12.6 ± 1.2	–	nd	4	6.9 ± 1.4	55.8 ± 7.9
Fenvalerate (II)	1††	10.7	–	nd	2	13.4 ± 3.2	–
Fenpropathrin (M)	3	10.8 ± 1.4	–	nd	3	12.4 ± 1.2	–
Cyhalothrin (U)	4	22.3 ± 4.3	–	nd	4	9.4 ± 0.5	84.7 ± 1.3
Cyfluthrin (U)	4	23.6 ± 6.2	–	nd	5	7.1 ± 0.5	95.9 ± 7.2
Tefluthrin (U)	3	3.8 ± 0.2	3	89.0 ± 18.9	3	4.3 ± 0.3	–
Bifenthrin (U)	4	3.9 ± 0.5	–	nd	4	4.0 ± 0.6	–

* All compound assayed at 100 µm except deltamethrin at 10 µm; classification indicated in parenthesis: I, Type I; II, Type II; M, Mixed; U, Unclassified.

† Time constant (ms) for the activation of pyrethroid‐modified channels based on reconstructed currents.

‡ Time constant (ms) for the inactivation of pyrethroid‐modified channels based on reconstructed currents.

§ Time constant (ms) for the fast (τ1) and slow (τ2) components of tail current decay.

** No detectable inactivation of pyrethroid‐modified current during a 40‐ms depolarizing pulse.

†† Only a single experiment with fenvalerate yielded a reconstructed current that could be fit to the first‐order activation model.

The VGSC has been established as an important target site for pyrethroids and, although there is only a single form in insects, there are nine α‐isoforms in mammals.^26^ Of these nine, six are sensitive to blockage by TTX at submicromolar levels (Nav1.1 to Nav1.4, Nav1.6 and Nav1.7), as is the insect channel. TTX‐sensitivity in insects is one reason for the current interest in the effects of pyrethroids on TTX‐sensitive VGSC isoforms in mammals. Although the Nav1.8 is insensitive to TTX, it is one of the most sensitive to pyrethroids among rat VGSC isoforms.^24^, ^25^ Further, this channel is absent from the mammalian brain, being found predominantly in dorsal root ganglion cells in the spinal cord. However, it has been studied in multiple systems, including rat Nav1.8 expressed in Xenopus oocytes^24^, ^25^ and human Nav1.8 in CHO cells, as shown in this report. The effects of pyrethroids on rat and human Nav1.8 channels showed similarities. Type I pyrethroids caused a large prolongation of inactivation and a large increase in relatively short‐lived tail current (deactivation) at the end of a depolarizing pulse. However, Type II pyrethroids had a modest effect on inactivation, similar to bifenthrin, combined with causing a slowly declining tail current. On human Nav1.8 channels, the assessment of efficacy (size of tail current) showed bifenthrin clustered with Type II pyrethroids (low efficacy), compared with Type I pyrethroids having high efficacy. The assessment of sensitivity or potency (EC_50_) also showed bifenthrin clustering with Type II pyrethroids (high potency) *vs*. Type I pyrethroids with low potency.

In an earlier rat study, Multi‐Dimensional Scaling (MDS) plots were made of clinical signs representing the Type II (CS) syndrome or Type I (T) syndrome against measures of activity on the rat Nav1.8 channel.^25^ Type I pyrethroids formed a cluster on the left‐hand side of the plot and the Type II, on the right‐hand side of the plot. Bifenthrin was positioned between these two groups of pyrethroids, for both sets of clinical signs. Fenpropathrin, also positioned between the two groups, is known to be a mixed Type I/II pyrethroid in a variety of systems.^13^, ^14^, ^15^ Esfenvalerate, considered to have a Type II pyrethroid mode of action in many systems was also positioned between the Type I and II pyrethroids.^25^ However, in the rat ASR test, fenvalerate effects were similar to Type I (increased amplitude with no effect on latency, Tmax).^27^, ^28^, ^29^ These are unlike Type II pyrethroid effects on ASR, where decreased amplitude was combined with increased latency.^28^ Using MDS scaling, in a similar manner, pyrethroid effects on voltage‐gated calcium channels (VGCCs) in rat brain synaptosomes were plotted against CS scores and T scores.^25^ As for the rat Nav1.8, Type I pyrethroids clustered to the left in the figure in reference^25^ and Type II to the right, helping to define Type I and Type II pyrethroid effects, with bifenthrin in between the two groups.^30^

The relative sensitivity of isolated rat and human Nav isoforms to pyrethroids has previously been studied.^31^ Rat and human Nav1.3 channels, expressed in *Xenopus* oocytes under voltage clamp, were incubated with tefluthrin (Type I)^32^ at 100 µm. The increase in sodium inactivation was much greater in the rat than in the human Nav1.3, when measured at 19–21 °C. It remains to be seen whether other VGSC isoforms show reduced pyrethroid sensitivity in human *vs*. rat isoforms when tested in the same expression system.

Studies on cortical neuron preparations, in culture^33^, ^34^ show that Type II pyrethroids increase Na^+^ influx (mouse) or reduce mean firing rate (rat). It is unexpected that these effects are opposite in direction. Moreover, in mouse neurons, Type I pyrethroids (including bifenthrin) generally had little or no effect on Na^+^ influx. However, a Type I pyrethroid (permethrin) antagonized competitively the Na^+^ influx increase of a Type II pyrethroid (deltamethrin). Using a Schild plot, the affinity value for permethrin was 4.43 µm.^33^ In rat cortical neurons, all (four) Type II pyrethroids (cypermethrin, deltamethrin, β‐cyfluthrin and esfenvalerate) reduced mean firing rate, whereas a Type I pyrethroid (permethrin) increased mean firing rate. It thus appears that Type I and II pyrethroids have qualitatively different effects on these TTX‐sensitive VGSC assays, making it confusing to describe Type I and II effects as additive, thus implying a common mode of action.

Bifenthrin dosing in rats resulted in profuse salivation, normally associated with Type II pyrethroids, but also with a small number of Type I or mixed pyrethroids. In the bifenthrin DNT study, there was a greater effect on clinical signs in dams than pups, but this could be a result of greater internal exposure in dams than pups i.e. a PK effect, rather than being a result of greater dam sensitivity i.e. a PD difference. Clinical signs recorded included both tremors (Type I) and clonic convulsions (Type II). The effects on the ASR in PND20 females, after early‐life dietary exposure, numerically decreased amplitude and significantly increased latency (Tmax), are typical of Type II pyrethroids in acute, oral gavage studies in adults;^27^, ^28^, ^29^ Dietary exposure to bifenthrin would result in a lower blood Cmax than after oral gavage dosing. On the other hand, Type I pyrethroids (and DDT) result in an increase in the ASR amplitude with no effect on latency in adult rats. In conclusion, the results of the ASR study suggested bifenthrin could arguably be classified as a mixed Type I/II pyrethroid. In this context, it could be relevant to point out that the α‐ethynyl analog of fenvalerate (α‐CN‐containing, Type II) showed hybrid clinical signs in the rat of tremors with salivation.^13^ Taken together, the results from various *in vivo* and *in vitro* comparative studies suggest that bifenthrin could be considered a mixed Type I and Type II pyrethroid. This could have significant consequences in conducting cumulative risk assessments for pyrethroids.

## 5 CONCLUSIONS

Bifenthrin was evaluated in a rat developmental neurotoxicity (DNT) study and in human VGSC expressed in CHO cells. In the DNT study, bifenthrin effects showed similarities to Type I and II pyrethroids. The combined effects of bifenthrin on ASR amplitude and latency are consistent with Type II *vs*. Type I pyrethroids. Effects on human Nav1.8 tail currents showed both Type II and I effects. Data described in literature from *in vitro* and *in vivo* studies also show mixed Type I and II effects for bifenthrin. Molecular modeling of the bifenthrin ester indicates that the *o*‐Me group may occupy a similar space to the α‐CN group of cypermethrin/fenpropathrin. Overall, data from a variety of pyrethroid mode action studies show that bifenthrin acts as a mixed Type I/II pyrethroid.

## Supporting information


**Appendix S1 Supplementary materials and methods**
**Figure S1. Voltage protocols.** Pre‐conditioning depolarizing steps (*Voltage Protocol A*) used to enhance hNav1.x sensitivity to local anesthetic‐type blockers (e.g. Lidocaine) and certain activators (e.g. Veratridine). To detect compounds affecting Nav channels kinetics of deactivation the *voltage protocol B* was used. The currents elicited by TP1b and TP2b, respectively, measure effects on deactivation and inactivation kinetics.
