# Supplementary material for: Pyrethroid neurotoxicity studies with bifenthrin indicate a mixed Type I/II mode of action
Source: Pest Manag Sci. 2019 Jan 30;75(4):1190–7. doi: 10.1002/ps.5300 (PMC6590159; doi:10.1002/ps.5300)
Supplement: Supplementary file 1 — Appendix S1 Supplementary materials and methods Figure S1. Voltage protocols. Pre‐conditioning depolarizing steps (Voltage Protocol A) used to enhance hNav1.x sensitivity to local anesthetic‐type blockers (e.g. Lidocaine) and certain activators (e.g. Veratridine). To detect compounds affecting Nav channels kinetics of deactivation the voltage protocol B was used. The currents elicited by TP1b and TP2b, respectively, measure effects on deactivation and inactivation kinetics. [file PS-75-1190-s001.docx]

**Supplementary Information for Section 2.3 - Bifenthrin effects on the human VGSC Nav1.8/β3 in CHO cells**.

**MATERIALS AND METHODS**

**Cell Lines**

Cell lines stably expressing human Nav1.1 (Chinese Hamster Ovary tetracycline-inducible cells (CHO-T); SCN1A; Catalog #CT6178), human Nav1.2 (CHO-T; SCN2A; Catalog #CT6010), human Nav1.3 (CHO-T; SCN3A; Catalog #CT6157), human Nav1.4 (CHO); SCN4A; Catalog #CT6005), human Nav1.5 (CHO; SCN5A; Catalog #CT6007), human Nav1.6 (CHO-T; SCN8A; Catalog #CT6158), human Nav1.7 (CHO-T; SCN9A; Catalog #CT6003), human Nav1.8/β3 (CHO-T; SCN10A/ SCN3B; Catalog #CT6011) ion channels were constructed as described previously^1^. The cells were maintained in 100-mm cell culture dishes in Ham’s F-12 CHO media supplemented with 10% fetal bovine serum, 100 U/mL of penicillin G sodium, 100 mg/mL of streptomycin sulfate, and the appropriate selection antibiotics.

Prior to experiments, the cells were passed in a medium free of selection antibiotics. Expression was induced with tetracycline 16–24 h before recording. Cell density was 50%–70% confluent at the time of harvest. Cells were harvested by washing twice with 15–20 mL of Hank’s Balanced Salt Solution (HBSS) and treatment with Accutase (Innovative Cell Technologies, San Diego, CA) solution for 20 minutes. Detached cells were transferred in a 15-mL conical tube and resuspended with addition of 10 mL of HBSS. Then the cells were pelleted at 500 g for 2.0 min, the supernatant was removed, and the cell pellet was resuspended in 10 mL of HBSS. The cell suspension was centrifuged again at 500 g for 2.5 min and the supernatant removed. Finally, the cell pellet was resuspended in 5mL of HEPES-buffered physiological saline (HB-PS): 137 mM NaCl, 4 mM KCl, 1.8 mM CaCl_2_, 1 mM MgCl_2_, 10 mM HEPES, and 10 mM glucose, pH adjusted to 7.4 with NaOH, and osmolarity adjusted to 295 ± 5 mOsm. The final cells dilution was 1.0^-6^ cells per mL, approximately.

**Solutions and Electrophysiological Procedures**

Chemicals used in a solution preparation were purchased from Sigma-Aldrich (St. Louis, MO) and were of ACS reagent grade purity or higher. Stock solutions of test articles were prepared in dimethyl sulfoxide (DMSO) and stored frozen. Each test article formulation was sonicated (Model 2510/5510; Branson Ultrasonics, Danbury, CT) at ambient room temperature for 20 min to facilitate dissolution. For experiments test article concentrations were prepared fresh daily by diluting stock solutions into extracellular solutions (HB-PS buffer) supplemented with 2 mM CaCl_2_. The final solution composition was 137 mM NaCl, 4 mM KCl, 3.8 mM CaCl_2_, 1 mM MgCl_2_, 10 mM HEPES, and 10mM glucose, pH adjusted to 7.4 with NaOH. All test and control solutions contained 0.3% DMSO. The test article formulations were prepared in 384-well compound plates using an automated liquid handling system (Cyclone, Caliper). The internal HEPES-buffered solution consisted of 90 mM CsF, 50 mM CsCl, 2 mM MgCl_2_, 2.5 mM EGTA, and 10 mM HEPES, pH 7.2 adjusted with CsOH. A stock solution of Amphotericin B (perforating agent) was prepared in DMSO (30 mg/mL) and added to the internal solution at a final concentration of 100 μg/mL.

Recordings were performed on IonWorks Barracuda™ system (Molecular Devices) in Population Patch-Clamp™ (PPC) mode as previously described^2^. The extracellular solution was loaded into the PPC plate wells (11 μL/well) and a cell suspension was added into the wells (9 μL/well). After establishment of a whole-cell configuration (7-min perforation), membrane currents were recorded by IWB on-board patch clamp amplifiers.

*Test Article Administration:* Test article concentrations were applied to naïve cells (4 wells/ concentration). Each application consisted of addition of 20 μL of 2X concentrated test article solution to the total 40 μL of final volume of the extracellular well of the PPC planar electrode. Duration of exposure to each test article concentration was five (5) minutes.

*Voltage Stimulus:* effects of test articles Nav channels were measured using stimulus voltage patterns shown in Figure 1S. The pulse patterns was repeated before (baseline) and for five (5) minutes after test article addition. Peak current amplitudes were measured for test pulses TP1a (tonic effects) and TP20a (use-dependent (10 Hz) effects); to characterize Pyrethroids effects on Nav channel deactivation the tail currents were measured after the end of test pulses TP1b (mean current on 10-ms interval). The peak currents elicited by TP2b were used to assess the full channel activation in control and after application of a test article.

*Voltage Protocol A*

**
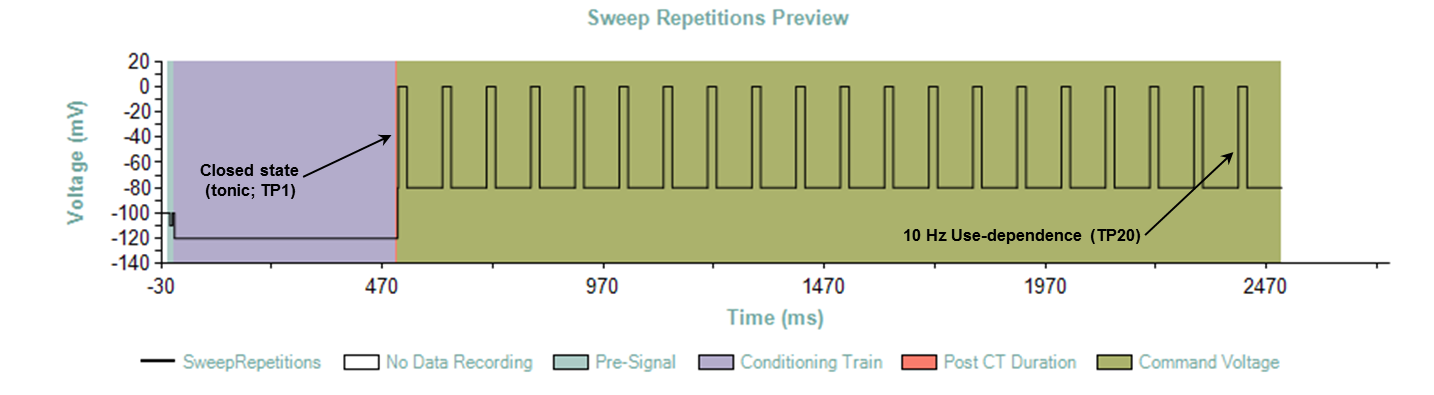
**

*Voltage Protocol B*

**
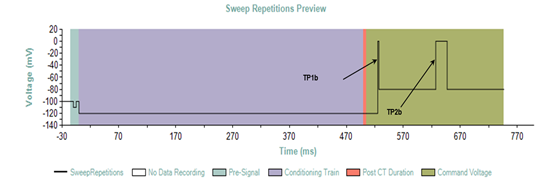
**

**Figure 1S. Voltage protocols.** Pre-conditioning depolarizing steps (*Voltage Protocol A*) used to enhance hNav1.x sensitivity to local anesthetic-type blockers (e.g., Lidocaine) and certain activators (e.g. Veratridine). To detect compounds affecting Nav channels kinetics of deactivation the *voltage protocol B* was used. The currents elicited by TP1b and TP2b, respectively, measure effects on deactivation and inactivation kinetics.

**Data Analysis**

Perythroids-produced activation of Nav channel tail currents was analyzed with the voltage protocol *B* (Figure 1S) and calculated as:

% Activation = (I_TP1b Tail, TA_ / (I_TP2b Peak, baseline_) x 100%;

Where, I_TP1b Tail, TA_ was the inward Na^+^ tail current (the mean current on 10-ms interval) elicited by the TP1b in the presence of a test article, I_TP2b Peak, Baseline_ was the baseline peak current elicited by the TP2b (pre-test article).

Concentration–response data were fitted to an equation of the following form:

% Activation =% VC + ((% 100 -% VC)/ (1 + ([Test]/EC_50_)^N^)),

where [Test] was the concentration of agonist, EC_50_ was the concentration of agonist producing half-maximal activation, N is the Hill coefficient, % VC was the percentage of the current signal at addition (the averaged current at the DMSO vehicle control addition), and % Activation is the percentage of the current signal elicited at each concentration of agonist. Nonlinear least squares fits were solved with the XLfit add-in for Excel 2003 (Microsoft).

**Acceptance Criteria**

Individual well data were filtered according to electrical criteria and the experiments were accepted based on plate level acceptance criteria.

Well Acceptance Criteria:

- Seal Resistance (baseline): Rseal ≥ 500 MOhm;
- Current amplitude(baseline): inward peak current ≥ 0.2 nA

Plate Acceptance Criteria:

- Z’ factor (assay sensitivity) ≥ 0.5.

Z’ factor for each experiment was calculated as:

Z’ = 1 – ((3x SDVC + 3x SDPC)/ABS (MeanVC – MeanPC),

Where MeanVC and SDVC were the Mean and Standard Deviation values for a vehicle control, MeanPC and SDPC were the Mean and Standard Deviation values for a positive control (3 mM Lidocaine).

**References for Supporting Information**

1. Wible BA, Kuryshev YA, Smith SS, Liu Z, Brown AM: An ion channel library for drug discovery and safety screening on automated platforms. Assay Drug Dev Technol (2008); 6:765–780.
2. Kuryshev, YA, Brown AM, Duzic E, Kirsch GE: Evaluating state dependence and subtype selectivity of calcium channel modulators in automated electrophysiology assays. Assay and drug development technologies (2014); 12: 110-119.
